# γδ T cells in cancer: a small population of lymphocytes with big implications

**DOI:** 10.1002/cti2.1080

**Published:** 2019-10-10

**Authors:** Mathilde Raverdeau, Stephen P Cunningham, Cathal Harmon, Lydia Lynch

**Affiliations:** ^1^ School of Biochemistry and Immunology Trinity College Dublin Dublin Ireland; ^2^ Harvard Medical School Boston MA USA; ^3^ Brigham and Women's Hospital Boston MA USA

**Keywords:** antitumor immunity, CAR T‐cells, DOT cells, immunotherapy, tumor progression, γδ T cells

## Abstract

γδ T cells are a small population of mostly tissue‐resident lymphocytes, with both innate and adaptive properties. These unique features make them particularly attractive candidates for the development of new cellular therapy targeted against tumor development. Nevertheless, γδ T cells may play dual roles in cancer, promoting cancer development on the one hand, while participating in antitumor immunity on the other hand. In mice, γδ T‐cell subsets preferentially produce IL‐17 or IFN‐γ. While antitumor functions of murine γδ T cells can be attributed to IFN‐γ^+^ γδ T cells, recent studies have implicated IL‐17^+^ γδ T cells in tumor growth and metastasis. However, in humans, IL‐17‐producing γδ T cells are rare and most studies have attributed a protective role to γδ T cells against cancer. In this review, we will present the current knowledge and most recent findings on γδ T‐cell functions in mouse models of tumor development and human cancers. We will also discuss their potential as cellular immunotherapy against cancer.

## Introduction

**Figure 1:**
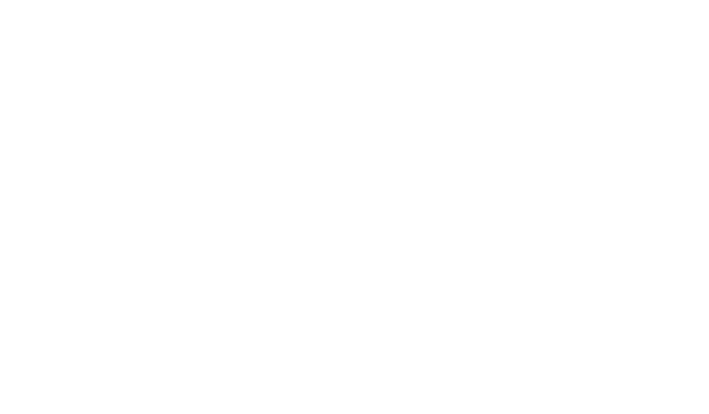
γδ T cells express an array of activating receptors for tumor cell recognition. Many of these mechanisms rely on the upregulation of stress ligands by tumor cells, including MICA/B (humans), Rae‐1/H‐60 (mouse) and ULBPs. γδ T cells also display an NK‐like phenotype in their expression of NCRs (NKp30, NKp44 and NKp46), particularly following activation. LFA‐1, lymphocyte function‐associated antigen 1; NKG2D, natural killer group 2 member D; PLZF, promyelocytic leukaemia zinc finger protein; Rae1, retinoic acid early inducible‐1; TCR, T‐cell receptor; TRAIL, TNF‐related apoptosis‐inducing ligand; ULBP, UL16‐binding proteins. * denotes expression on some clones only.

γδ T cells constitute non‐MHC‐restricted innate‐like T‐cell populations, poised to be activated rapidly within seconds to minutes, rather than days, and bridge the innate and adaptive immune systems.^1^ Although γδ T cells make up only a minor proportion of the CD3^+^ compartment in the circulation and most tissues, because of their rapid cytokine production following activation, they constitute an important first line of defence against infections and are important players in antitumor defence.^2^, ^3^ Innate recognition of tumor cells and subsequent activation of γδ T cells are mediated by a range of cellular and molecular determinants, including tumor‐derived stress ligands and cytokine signals (Figure 1). Despite their well‐documented innate properties, the adaptive features of γδ T cells are also essential in their development and function.^4^, ^5^, ^6^ Unlike αβ T cells, activation of γδ T cells through their TCR is generally thought not to be restricted to presentation of peptide by MHC molecules, although a human γδ T‐cell clone capable of recognising melanoma tumor antigens MART‐1 and gp100 in a MHC I‐restricted fashion was recently generated in an artificial experimental system.^7^ The identification of γδ TCR ligands and the antigen‐presenting molecules they recognise remains a long‐standing quest, although several candidates, linked to specific γδ T‐cell subsets, have been identified. Among these are non‐peptidic phosphorylated metabolites, or phosphoantigens (PAgs), recognised by human Vγ9Vδ2 T cells and expressed not only by pathogens but also by tumor cells (Figure 1).^8^ In addition, human and murine γδ T cells are thought to be capable of activation by cytokines, independent of TCR–cognate antigen recognition.

γδ T cells can be found in the circulation and in secondary lymphoid organs, but they are mainly resident in barrier tissues, such as the mucosae and the skin, and in adipose tissue. γδ T cells expressing specific Vγ and Vδ chains are enriched in particular locations within the body as illustrated in Table 1. This suggests that tissue‐specific factors trigger clonal selection, possibly as a result of infection, cytokine milieu or endogenous antigens, highlighting how little is known about factors controlling activation and expansion of human γδ T‐cell subsets. In mice, it is clear that two functional subsets of γδ T cells can be found, one producing IL‐17 and one producing IFN‐γ. These two subsets can be functionally defined based on differential surface expression of CD27. CD27 is a member of the TNF receptor family and binds to CD70. CD27 is expressed not only on activated lymphocytes but also on tumor cells. While CD27 is present on murine IFN‐γ^+^ γδ T cells, it is absent on the surface of the IL‐17^+^ γδ T cells.^9^ On the contrary, IL‐17 γδ T cells preferentially express CCR6 and the transcription factor PLZF, which is considered to confer innate‐like properties to lymphocytes.^10^, ^11^, ^12^, ^13^ Whereas murine γδ T cells acquire TCR‐dependent functional maturity during thymic ontogeny, human γδ thymocytes are functionally immature and instead acquire their effector functions in response to peripheral cytokine signals.^14^, ^15^, ^16^ Nevertheless, human thymic γδ T cells exhibit *de novo* expression of type 1 transcription factors T‐bet and eomesodermin, reflecting their capacity to rapidly differentiate into cytotoxic effectors producing IFN‐γ in response to cytokines IL‐2 and IL‐15.^15^ Unlike in mice, the γδ T‐cell compartment in humans cannot be functionally defined based on differential expression of CD27 and the functional distinction among the different subsets is less clear.^9^ Human γδ T cells can be divided into 3 main subsets based on TCRδ‐chain usage, Vδ1, Vδ2 and Vδ3, which does not allow for clear discrimination of their different effector functions. Interestingly, Vδ4^+^, Vδ5^+^ and Vδ6^+^ populations of γδ T cells have also been found in patients with diverse infections, but they remain rare and no commercially available antibodies exist for these subsets.^17^ Thus, most of the studies of human γδ T cells have focused on the Vδ1, Vδ2 and Vδ3 subsets. While tissue‐resident γδ T cells are mostly Vδ1^+^ (and probably Vδ3^+^, as they are sometimes described as Vδ1^−^Vδ2^−^), the majority of our current knowledge on the biology of human γδ T cells comes from blood‐circulating cells, which are mainly Vδ2^+^ (Table 1). Recent studies concerning the human γδ TCR repertoire have revealed distinct innate and adaptive roles for γδ T‐cell subsets, depending on TCRγ‐ and TCRδ‐chain usage. In cord blood, the Vδ1^+^ TCR repertoire is highly diverse and private, but undergoes postnatal clonotypic focusing throughout adulthood,^18^ as evidenced by the enrichment of discrete Vδ1^+^ clonotypes during cytomegalovirus (CMV) and human immunodeficiency virus (HIV)^19^ infection. Within the Vδ2^+^ subset exist highly clonal adaptive populations expressing a Vγ9^−^Vδ2^+^ TCR, which undergo differentiation and clonal expansion during acute CMV infection, in contrast to the innate‐like Vγ9^+^Vδ2^+^ TCR with limited recognition kinetics and CDR3 diversity.^20^ The Vδ2^+^ subset constitutes an heterogeneous population of cells, producing a range of pro‐inflammatory cytokines including IFN‐γ, IL‐17, TNF‐α, IL‐9, but also IL‐10 depending on the setting.^21^, ^22^, ^23^, ^24^

**Table 1:** The relative anatomical distribution and primary effector functions of different γδ T‐cell subsets in humans and mouse

Subset	Common γ‐δ chain pairings	Anatomical localisation	Context for the production of IFN‐γ or IL‐17	Other effector molecules
Mouse^a^				
Vγ1	Vγ1Vδ6.3/6.4	Liver, secondary lymphoid organs	IFN‐γ – cancer,^25^ viral infection^26^	TNF, IL‐4^27^
Vγ2	Undefined	Liver, lung (rare)	Undefined	Undefined
Vγ4	Vγ4Vδ4	Lung, liver, dermis, lamina propria, secondary lymphoid organs	IFN‐γ – cancer,^28^ IL‐17 – skin injury^29^	TNF, IL‐22^30^
Vγ5	Vγ5Vδ1 (DETC)	Epidermis	IFN‐γ – cancer, TLR signalling,^31^ NKG2D ligation^31^	TNF, IL‐22^32^
Vγ6	Vγ6Vδ1	Uterine epithelia, lung	IL‐17 – bacterial infection,^33^ cancer^34^	IL‐22^35^
Vγ7	Vγ7Vδ4/5/6	Gut epithelia	IFN‐γ – bacterial infection^27^	IL‐4, IL‐10^27^
Human				
Vδ1	Undefined	Gut epithelia, liver, dermis	IFN‐γ – cancer,^36^ IL‐17 – colorectal cancer^37^	TNF,^36^ IL‐10^23^
Vδ2	Vγ9Vδ2	Peripheral blood	IFN‐γ – cancer, phosphoantigen stimulation^8^IL‐17 – bacterial infection^24^	TNF,^21^ IL‐9,^22^ IL‐10^23^
Vδ3	Undefined	Gut epithelia, liver	IFN‐γ – glycolipids^38^	TNF, IL‐4^38^

Heilig and Tonegawa nomenclature.

While IFN‐γ‐producing γδ T cells are abundant in peripheral blood, IL‐17 production by human γδ T cells is rare at homeostasis. However, significant inflammatory insult such as that seen in some cancers and infections can polarise γδ T cells towards a type 17 phenotype.^24^, ^37^, ^39^, ^40^ A recent extensive study sequencing bulk transcriptomes of 18 000 human tumors revealed that, among all leucocytes present in the tumors, γδ T cells were most strongly associated with good prognosis.^41^ However, the computational approach used to characterise these cells has since been disputed.^40^ There have also been reports of γδ T cells having a potential tumor‐promoting role in various human malignancies,^37^, ^40^, ^42^ likely attributable to their functional plasticity in various inflammatory microenvironments, although determination of a direct immunosuppressive role for human γδ T cells *in situ* is difficult. Thus, although γδ T cells may still provide good prognostic and therapeutic value in human cancers, more research is required into understanding the balance between pro‐ and antitumor effector functions, and how this is regulated in the tumor microenvironment.

## γδ T cells in tumor immune surveillance and antitumor immunity

### Antitumor functions of murine γδ T cells

**Figure 2:**
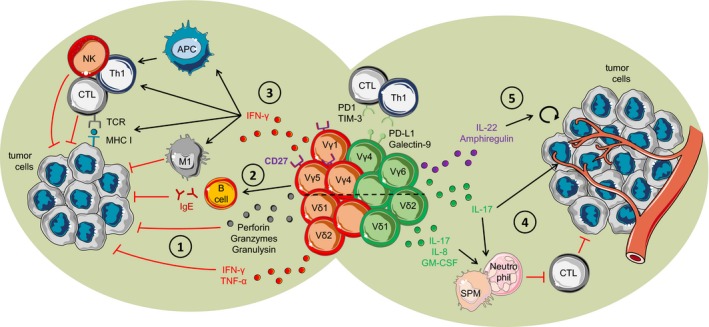
Pro‐ and antitumor effect of γδ T cells. (1) Antitumor immunity of γδ T cells by direct killing of tumor cells via perforin, granzymes, granulysin and cytokines. (2) Vγ5+ γδ T cells induce B‐cell class switching to autoreactive antitumor IgE. (3) IFN‐γ production by γδ T cells promotes the recruitment of NK, Th1 and CTLs and induces the differentiation of antitumor macrophages. Additionally, IFN‐γ enhances the presentation capacities of APCs and MHC I expression by tumor cells, while inhibiting pro‐tumor T helper cells. (4) γδ T cells producing IL‐17 promote angiogenesis and suppress antitumor CTL and Th1 cells. (5) Production of IL‐22 and amphiregulin by γδ T cells induces direct tumor cell proliferation. The dashed line separates mouse and human γδ T cells. γδ T cells depicted in red are the cells with antitumor functions, while γδ T cells depicted in green are the cells that promote tumor growth.

Initial studies performed in murine models of cancer have found protective roles for γδ T cells against tumor growth.^43^, ^44^ Several mechanisms, through which they mediate their antitumor effects, have been described, including not only direct killing of tumor cells mediated by cytolytic proteins or NKG2D‐dependent mechanisms, but also indirect effects mediated by their production of IFN‐γ, as illustrated in Figure 2. In this section, we summarise the current knowledge on the different antitumor functions attributed to murine γδ T cells.

Early studies on the protective role of γδ T cells in mice have been conducted in murine models of skin cancers, induced chemically or by subcutaneous transfer of melanoma or carcinoma cell lines. In all models, crucial roles for γδ T cells in antitumor immunity have been described, and studies have shown a NKG2D‐mediated mechanism by tissue‐resident Vγ5^+^ dendritic epidermal T cells (DETCs) as a main player in γδ T‐cell antitumor function.^43^, ^44^, ^45^, ^46^ DETCs are dendritic‐shaped γδ T cells, which express a largely invariant Vγ5^+^Vδ1^+^ TCR, and are considered to be a unique and unusual subset of γδ T cells, which restricts the extent of findings on these cells to other populations of γδ T cells. DETCs constitute the majority of T cells in the murine epidermis, but γδ T cells are far less abundant in human skin, and DETC equivalents are not present in humans, although evidence for antitumor function of skin γδ T cells also exists in humans.^47^ Interestingly, *in vivo* studies of skin cancer performed in γδ T‐cell‐deficient mice (TCRδ^*−/−*^ mice) did not allow for discrimination between DETCs and other populations of γδ T cells, and might have underestimated the role of dermal Vγ4^+^ γδ T cells or other subsets infiltrating the skin.^43^, ^44^ Indeed, TCRδ^*−/−*^ mice reconstituted with Vγ4^+^ γδ T cells had a restored antitumor response against B16 melanoma cells, which relied on IFN‐γ and perforin production, two important mediators in antitumor immunity by γδ T cells and other lymphocytes (Figure 2).^45^, ^48^, ^49^ Importantly, a protective role for γδ T cells in antitumor response in mice has been described in other models of cancer and notably in a spontaneous model of B‐cell lymphoma.^49^ While both perforin and IFN‐γ induce tumor cell death, IFN‐γ additionally promotes the recruitment and activation of other cytotoxic lymphocytes such as Th1 cells, NK cells and cytotoxic CD8^+^ T cells (CTLs), while inhibiting the differentiation of Th2, Th17 and Treg cells. IFN‐γ also drives a pro‐inflammatory phenotype in macrophages and enhances the antigen presentation capacities of professional APCs.^50^ Interestingly, IFN‐γ production by γδ T cells also enhances MHC I molecule expression at the surface of B16 melanoma cells, thereby promoting their recognition by CTLs (Figure 2).^51^ A recent study in a mouse model of gastrointestinal stromal tumor describes a protective role for γδ T cells mediated through the secretion of GM‐CSF. This cytokine promoted the maturation of CD103^+^ CD11b^−^ dendritic cells, which were associated with infiltration of effector CD8 T cells within the tumor.^52^

Tumor immune surveillance by activated murine γδ T cells has been linked to their surface expression of the C‐type lectin receptor NKG2D, ligands for which Rae‐1 and H‐60 (MICA and MICB in humans) are expressed at the surface of stressed cells.^43^, ^46^ In mice, DETCs together with Langerhans cells (epidermal dendritic cells) and tissue‐resident CD8^+^ memory T cells form a network integrated within the epidermis.^53^ Upregulation of Rae‐1 by epidermal cells induces activation and remodelling of DETCs from dendritic‐ to round‐shaped cells, leading to a reorganisation of the epidermal architecture. Rae‐1 upregulation also promotes expression of the activation marker CD69 on the DETCs within the epidermis and the killing of tumor cells through a NKG2D‐mediated pathway.^43^, ^46^ While expression of NKG2D ligands in humans is associated with better outcome in several types of cancers, NKG2D ligands are often internalised by tumor cells or secreted as soluble forms during immune evasion, but are promoted following exposure to different factors including chemotherapy.^54^ Interestingly, a recent study by Sheppard and colleagues has identified an unexpected tumor‐promoting role for NKG2D in a model of hepatocellular carcinoma. The authors proposed that, while NKG2D has evident antitumor function in early stages of cancer, it could exacerbate the pro‐inflammatory microenvironment of the tumor at later stages, leading to tissue damage and enhanced cell proliferation, which promoted tumor progression in the liver environment.^55^ While the authors did not look at the implication of γδ T cells in this process, these cells could nevertheless play a role, given their enrichment in the liver and their robust cytokine expression in response to many inflammatory signals.^56^

The engagement of the γδ TCR in tumor recognition and elimination by murine γδ T cells is also likely, and this is also true in humans.^57^ Indeed, Girardi *et al*.^43^ showed that incubation of murine DETCs with a γδ TCR‐blocking antibody resulted in impaired lysis of the PDV tumorigenic keratinocyte cell line. However, Dutta *et al*.^58^ have recently shown that blockade of γδ TCR with antibodies can induce apoptosis in those cells, which could account for the decrease in killing capacity observed. Recently, Crawford *et al*.^59^ showed that skin‐resident intraepithelial γδ T cells also induced a rapid adaptive immune response to chemically induced skin carcinogenesis by promoting class switching and secretion of high levels of protective IgE by B cells, indicating that the impact of γδ T cells on other cell types might be broader than expected (Figure 2).

### Role of human γδ T cells in antitumor immunity

#### The Vδ2^+^ subset

Vδ2^+^ γδ T cells are the predominant subtype in the blood, accounting for 2–5% of circulating CD3^+^ lymphocytes.^60^ These cells express a TCR with preferential pairing of Vδ2 and Vγ9 chains, and mediate effective antitumor immunity directly through cytotoxicity via perforin and granzymes, or indirectly through IFN‐γ and TNF production (Figure 2).^3^ Recognition of tumor cells by Vγ9^+^Vδ2^+^ T cells can occur through a host of cell surface receptors for self and non‐self ligands, including TCR recognition of tumor antigen and stress ligand receptors. These include NKG2D, FCγIII (CD16), FasL, TRAIL and DNAM‐1 (CD226).^61^, ^62^, ^63^, ^64^, ^65^ Vγ9^+^Vδ2^+^ T cells recognise tumor‐derived phosphorylated prenyl metabolites in a TCR‐dependent manner, which may accumulate intracellularly as a by‐product of dysregulated tumor metabolism (Figure 1). One well‐studied PAg, isopentenyl pyrophosphate (IPP), can accumulate in cancer cells as a result of the elevated metabolic flux through the mevalonate pathway of cholesterol biosynthesis.^21^, ^28^, ^66^ These non‐peptidic antigens are not presented in the context of classical MHC and are instead presented through a non‐polymorphic type I transmembrane protein called butyrophilin 3A1 (BTN3A1). BTN proteins of the immunoglobulin (Ig) superfamily consist of a B30.2 intracellular domain and two extracellular Ig domains.^67^ The mechanism of activation of Vγ9^+^Vδ2^+^ T cells by BTN3A1‐bound PAg remains controversial, although it is thought to be triggered by initial intracellular binding of PAg to a positively charged surface pocket within the intracellular B30.2 domain.^68^, ^69^, ^70^, ^71^ The resultant conformational change within BTN3A1 has been proposed to confer recognition by the Vγ9Vδ2 TCR in an ‘inside out’ signalling mechanism whereby surface BTN3A1 is sensitive to the intracellular concentration of prenyl pyrophosphate metabolites.^72^, ^73^ These non‐MHC‐restricted, innate‐like recognition kinetics of Vγ9^+^Vδ2^+^ T cells are an attractive candidate for cancer immunotherapy and have been targeted in clinical settings using aminobisphosphonate drugs. Aminobisphosphonates are clinically approved potent inhibitors of the mevalonate pathway, thereby not only promoting direct antitumor effects but also leading to a build‐up in endogenous isoprenoid metabolites. Zoledronate is an aminobisphosphonate drug that directly inhibits farnesyl pyrophosphate synthase (FPPS), an enzymatic mediator of the mevalonate pathway, leading to a build‐up in endogenous IPP.^74^ This, in combination with mitogenic IL‐2, induces activation and proliferation of type 1 cytotoxic effector γδ T cells with antitumor potential producing IFN‐γ, TNF, perforin and granzymes.^75^ Thus far, all clinical trials using γδ T cells as an autologous cellular therapy for cancer have focused on *ex vivo* or *in vivo* activation and expansion of Vγ9^+^Vδ2^+^ T cells with aminobisphosphonates, with satisfactory safety profiles observed.^76^, ^77^ This highlights the need for a better understanding of how Vγ9^+^Vδ2^+^ T cells become activated in cancer, how their effector functions are regulated and how this may be exploited for therapeutic gain in cancer immunotherapy.

#### The Vδ1^+^ subset

Vδ1^+^ T cells are a minor population in the blood but represent the predominant tissue‐resident population of γδ T cells.^57^ These cells are mainly found at mucosal sites such as the dermis and intestinal epithelia where they can comprise 20–50% of the tissue‐resident lymphoid compartment.^3^ Unlike their Vδ2^+^ counterparts, Vδ1^+^ γδ T cells do not often preferentially pair with a specific Vγ chain (although clonal expansion can be seen in some organs, which can be different among individuals). Vδ1^+^ γδ T are not activated by PAgs, but can display an NK‐like phenotype in their expression of natural cytotoxicity receptors, (NCRs) NKp30, NKp44 and NKp46, depending on the protocol used to expand them.^62^ Although a unique ligand for the Vδ1^+^ TCR has yet to be identified, recent studies have elucidated some cognate TCR recognition properties of Vδ1^+^ T cells. A crystallographic study revealed sequential recognition kinetics of the MHC class I homologue MICA by NKG2D and Vδ1^+^ TCR, thereby providing both TCR and costimulatory signals from the same ligand.^65^ Some Vδ1^+^ cell lines have been reported to recognise the lipid antigen α‐galactosylceramide (α‐GalCer) presented by CD1d.^78^, ^79^, ^80^ Furthermore, Vδ1^+^ TCR‐mediated recognition of glycolipids presented in the context of CD1c facilitates target cell lysis, Th1 cytokine production and dendritic cell maturation by Vδ1^+^ T cells (Figure 1). Indeed, as with αβ T cells, TCR‐mediated recognition of host stress ligands by γδ T cells may require costimulatory signals. This is exemplified by the TCR‐mediated recognition of endothelial protein C receptor (EPCR) expressed on CMV‐infected endothelial cells by a γδ T‐cell clone bearing a Vγ4Vδ5 TCR, which required CMV‐induced upregulation of ICAM‐1 by target cells for an optimal response.^81^ Similar to Vδ2^+^ cells, Vδ1^+^ γδ T cells induce tumor cell death through soluble cytotoxic machinery (perforin, granzymes and granulysin) and cytokine secretion (IFN‐γ and TNF). The cytolytic function of Vδ1^+^ T cells has been shown for a range of haematological and solid malignancies, including acute lymphoblastic leukaemia (ALL), acute myelogenous leukaemia (AML), B‐cell chronic lymphocytic leukaemia (B‐CLL), neuroblastoma, melanoma and pancreatic, lung and colorectal cancers (CRC).^36^, ^57^, ^60^, ^82^, ^83^, ^84^ Vδ1^+^ T cells seem to outclass Vδ2^+^ cells in most *in vitro* and in several *in vivo* pre‐clinical cancer models in terms of cytotoxicity and durability,^57^, ^85^ which may have important implications in the development of next‐generation γδ‐based immunotherapies. One advantage Vδ1^+^ cells may have for use in immunotherapy is their resistance to activation‐induced cell death (AICD),^86^ which has posed significant problems in clinical trials following chronic stimulation of Vγ9^+^Vδ2^+^ T cells with aminobisphosphonate drugs.^77^ Although the cytotoxic capacity of both Vδ1^+^ and Vγ9^+^Vδ2^+^ T cells makes them attractive targets for the development of next‐generation immunotherapies, a broader understanding of how these effector functions are regulated and how they may be polarised towards a pro‐tumor phenotype, and whether, like conventional T cells, they become inhibited and exhausted in the tumor microenvironment, is required.

## γδ T cells as drivers of tumor growth

### Pro‐tumor function of murine γδ T cells

Studies performed in mouse models indicate that pro‐tumor functions of γδ T cells can be largely attributed to the IL‐17^+^ cells (Figure 2). This is in line with the majority of reports on IL‐17 production by other innate and adaptive immune cells, although the impact of IL‐17 on tumor growth might depend on the type of cancer studied.^87^ We and others have found that IL‐17^+^ γδ T cells are enriched not only in a variety of murine solid tumor models induced by implantation of tumorigenic cells, but also in spontaneous models of HPV‐related carcinogenesis and breast cancer models, for which they are associated with metastasis.^34^, ^88^, ^89^, ^90^, ^91^, ^92^ In murine models of ovarian, pancreatic and lung cancers, IL‐17‐producing γδ T cells in tumors were highly proliferative and displayed an activated phenotype.^34^, ^89^, ^93^ They induced angiogenesis and the recruitment of neutrophils, generally associated with poor prognosis in cancer.^92^, ^93^ Indeed, neutrophils secrete different tumor‐promoting agents, such as growth factors, metalloproteinases (MMPs), neutrophil elastase (NE) and reactive oxygen species (ROS), which directly enhance tumor growth and invasion, promote angiogenesis and suppress antitumor immune cells. Nevertheless, neutrophils display phenotypical and functional plasticity depending on the tumor microenvironment, and have been found to also contribute to antitumor immune response, notably through antibody‐dependent cellular cytotoxicity (ADCC) and recruitment of other immune cells.^94^ IL‐17‐producing γδ T cells also promoted the recruitment of immunosuppressive neutrophils and small peritoneal macrophages, which inhibit CTL response and enhance tumor growth.^34^, ^88^, ^90^, ^93^ In a model of pancreatic ductal adenocarcinoma (PDA), IL‐17^+^IL‐10^+^ γδ T cells were also directly suppressive of T‐cell responses. Here, IL‐17^+^ γδ T cells expressed the checkpoint inhibitors PD‐L1 and Galectin‐9, both of which prevented the activation of αβ CD4^+^ and CD8^+^ T cells, indicating that γδ T cells can directly inhibit adaptive antitumor immunity (Figure 2).^89^ A direct role of IL‐17^+^ γδ T cells on tumor cell proliferation is also possible as IL‐17^+^ γδ T cells from lung tumors expressed IL‐22 and amphiregulin, both of which can directly promote tumor cell proliferation (Figure 2).^93^ Nevertheless, as IL‐17‐producing γδ T cells are rarely found in humans at steady state, further studies are needed to fully grasp the relevance of these findings for human cancers.

The tumor microenvironment provides favorable conditions for the enrichment of IL‐17‐producing γδ T cells, notably through enhanced levels of the cytokines IL‐1β, IL‐6, IL‐23 and IL‐7, which favor CD27^−^ γδ T‐cell survival and promote IL‐17 expression.^34^, ^88^, ^92^

Jin *et al*.^93^ showed that in a spontaneous model of lung adenocarcinoma, tumor development alters the local microbiota, which induces the production of IL‐1β and IL‐23 by myeloid cells resulting in highly proliferative tissue‐resident IL‐17^+^ Vγ6^+^Vδ1^+^ γδ T cells. Interestingly, the IL‐17 production in γδ T cells via IL‐1β axis is also described in promoting tumor metastasis in a spontaneous model of breast cancer metastasis.^92^ Interestingly, IL‐1β and IL‐6 additionally drive the expression of NOS2, associated with tumor evasion, in pro‐tumorigenic γδ T cells.^95^ IL‐17^+^ γδ T‐cell recruitment is supported by tumor chemokine secretion, such as CCL2/MCP‐1, a molecular target for anticancer therapy and ligand for CCR2, which is highly expressed on tumor‐infiltrating γδ T cells.^89^, ^91^, ^96^, ^97^, ^98^ The chemokine receptor CCR6, involved in the trafficking of IL‐17^+^ cells to tissues at steady state, is also expressed by IL‐17^+^ γδ T cells in the tumor bed of PDA and hepatocellular carcinoma.^89^, ^90^ Indeed, CCR6 and its ligand CCL20 are associated with tumor progression in models of CRC and pancreatic cancer.^99^, ^100^ Interestingly, in other models, recruitment of IL‐17^+^ γδ T cells to the subcutaneous B16 melanoma tumors and HPV‐induced skin lesions, respectively, is associated with a downregulation of CCR6 expression,^91^, ^96^ indicating that the environmental setting in which the tumor develops might influence the phenotype of the immune cells recruited.

Intrinsic metabolic pathways are another parameter that may influence the recruitment and survival of γδ T cells within the tumor bed. In fact, as cancer progresses, tumor cells override lymphocytes in competition for nutrients, especially glucose, which is essential for T‐cell effector functions. Thus, nutrient availability might favor or limit the survival of particular immune cells. Our unpublished work suggests that CD27^−^ and CD27^+^ γδ T cells have different metabolic requirements, which might partially explain the enrichment of the IL‐17^+^ subset over the IFN‐γ^+^ one in the tumor, observed in a number of cancer models. In addition, tumor cells and other cells infiltrating the tumor niche express enzymes and excrete products, which can inhibit normal T‐cell metabolism. For instance, in models of hepatocellular carcinoma and peritoneal B16 tumor, tumor‐infiltrating IL‐17^+^ Vγ6^+^ γδ T cells express low amounts of the antioxidant glutathione, which make them highly susceptible to ROS produced by tumor‐associated neutrophils.^101^ These recent insights of the effect of metabolic state of the tumor microenvironment on the promotion of pro‐ or antitumor immune cells require further investigation.

### Pro‐tumor function of human γδ T cells

One potential caveat of the functional plasticity and innate response kinetics of γδ T cells is their susceptibility to polarisation by a particular inflammatory milieu. Although human γδ T cells rarely produce IL‐17, several groups have reported an elevated frequency of IL‐17^+^ γδ T (γδT17) cells in response to a combination of Th17‐polarising cytokines IL‐1β, IL‐6, IL‐23 and TGF‐β in some disease settings.^24^, ^39^ Many of these cytokines are elevated in the tumor microenvironment of certain cancers, and indeed, there have been some reports of IL‐17‐producing γδ T cells having a pro‐tumor role in various human malignancies.

The first report of IL‐17‐producing γδ T cells having a pro‐tumorigenic role in humans was reported by Wu *et al*.^37^ in patients with CRC. They showed that breach of the gut epithelial barrier by tumor dysplasia induced an influx of commensal microbial products, resulting in the accumulation and activation of IL‐23‐producing inflammatory dendritic cells. This was sufficient to induce γδT17 polarisation of Vδ1^+^ intraepithelial lymphocytes, with γδ T cells identified as being the main cellular source of IL‐17 in human CRC. Production of IL‐8 and GM‐CSF by γδ T cells resulted in an influx of immunosuppressive neutrophils, which have well‐established pro‐tumor roles in an array of cancer types in both humans and mice.

McAllister *et al*.^102^ used a murine model of pancreatic intraepithelial neoplasia (PanIN), a histological precursor of PDA, to show that the oncogene Kras can induce the expression of IL‐17 receptors on PanIN cells and infiltration of IL‐17^+^ lymphocytes into pancreatic stroma. Within the pancreatic tumor microenvironment exists an abundance of type 17‐polarising cytokines such as IL‐6 and TGF‐β.^103^ They showed an increase in the frequency of RORγt^+^ cells in PanIN lesions, primarily produced by Th17 (10% IL‐17^+^) and γδ (50% IL‐17^+^) T cells. It has later shown that human PDA consists of a unique inflammatory infiltrate, with γδ T cells making up to 75% of infiltrating T cells,^89^ although Gunderson *et al*.^104^ have reported a much lower proportion of γδ T cells in the PDA inflammatory infiltrate (< 5%). Using a transgenic murine model of PDA, they identified a substantial population of γδ T cells, which produced IL‐10 and IL‐17 and restrained αβ T‐cell activation through expression of immune checkpoint ligand PD‐L1. Although the role of IL‐17 in human pancreatic tumorigenesis remains uncharacterised, ablation of γδ T cells resulted in enhanced αβ T‐cell tumor infiltration with superior antitumor effector function in TCRδ^−/−^ mice, perhaps highlighting the need for a better understanding of how γδ T‐cell anticancer function is regulated in the tumor microenvironment.

A pathological role has also been described for γδT17 cells in human gallbladder cancer (GBC), with an increased frequency of γδ TCR^+^ cells in the blood and TIL of patients with GBC.^40^ Here, γδT17‐derived IL‐17 induced expression of vascular endothelial growth factor (VEGF) and other pro‐angiogenic factors by GBC cells, facilitating tumor growth and survival. A common feature of the cancer types for which a pro‐tumorigenic role of γδ T cells has been described is their resistance to conventional chemotherapeutic treatments and poor 5‐year survival rates, exemplifying the need for alternative therapies. With the recent clinical success of immunotherapies such as checkpoint blockade and chimeric antigen receptor (CAR) T cells, an argumentative case can be made for targeting γδ T cells; however, the factors that govern the pro‐ versus anticancer phenotype in the tumor microenvironment must first be further explored.

## Applications of γδ T cells in immunotherapy

Immunotherapy is a rapidly expanding and diversifying field of clinical oncology, which has shown unprecedented success in the clinic. The emergence of immune checkpoint inhibitors and CAR T‐cell technology has revolutionised the treatment of malignancy. However, the efficacy of these treatments is limited, for the most part, to haematological neoplasms and solid tumors with high mutational burdens (e.g. metastatic melanoma and MSI‐high colon cancer).^105^ While current T‐cell‐based therapies have shown great success in the clinic, several pitfalls in their use still persist. Checkpoint inhibitors are only effective in a minority of patients, acquired resistance and tumor relapse with resistant clones is an increasingly worrying problem. The time and expense involved in the expansion and conversion of patient cells to CAR T‐cell products means there is a limited treatment window available to patients with advanced disease. In some cases, this expansion protocol fails completely, leaving few options for further treatment. Similar to checkpoint therapy, the use of CAR T‐cells in solid tumors has proven disappointing. This is believed to be in large part by difficulty in drawing them to the affected tissues. Homing to effected tissues requires the expression of a range of chemokine receptors and adhesion molecules, which are not normally expressed by peripheral blood T cells.^106^ Successful elimination of tumors is dependent on the persistence of transferred T cells, which can become exhausted. Conversely, some patients suffer detrimental side effects, such as autoimmune colitis and cytokine release syndrome. These side effects can even result in increased morbidity and mortality.^107^ Therefore, an off‐the‐shelf cellular immunotherapy is an attractive proposition. Innate immune cells, such as γδ T cells and NK cells, appear to have an improved safety profile with minimal off‐target effects.^108^ Furthermore, since these cells are not MHC‐restricted cell products can be prepared from a pool of healthy donors and expanded, reducing the costs and unpredictability associated with rapid expansion of patient‐derived products. The innate nature of γδ T cells and their ability to recognise a wide range of tumors makes them potentially excellent candidates for cellular therapy.

A pan‐cancer analysis of the TCGA database identified γδ T cells as the strongest immune prognostic available in solid tumors.^41^ However, the analysis showed wide variability in the infiltration of tumors by γδ T cells. In addition, the computational algorithm used to deconvolute these tumor microarrays, CIBERSORT, has then shown to inaccurately distinguish γδ T cells from other lymphoid populations.^109^ This computational‐based identification was later optimised by Tosolini *et al*.,^109^ allowing for more accurate assessment of γδ TILs from bulk tumor transcriptomes. Moreover, γδ T cells are diverse and often plastic so identifying the most suitable subset and maintaining this phenotype *in vivo* remains a challenge to be addressed in coming years. For example, the presence of IL‐17‐producing γδ T cells in colon cancer has been associated with poor prognosis.^37^, ^110^ Interestingly, though a pan‐cancer analysis of the TCGA database identified a combined Th1/Th17 immune signature as the most beneficial for patient survival, this group showed the most pronounced Th17 gene signature but appeared balanced by the presence of a Th1 response.^111^ This study requires further dissection to determine the relative contribution of Th1 and Th17 genes to this signature. IL‐17 has previously been considered pro‐tumorigenic, with many IL‐17‐mediated diseases eventually leading to malignancy. However, this study indicates that IL‐17 in context of a Th1 response may be beneficial, but the source and localisation of IL‐17 production cannot be identified in current transcriptomic data sets with reasonable certainty. Therefore, this question may benefit from a new approach, and using single‐cell transcriptomic analysis to identify the source of this potentially beneficial IL‐17 is worth investigation. Homology between murine and human γδ T‐cell subsets is poor and makes translation of murine studies to humans a difficult proposition.

Vδ1^+^ T cells make up a small proportion of the circulating γδ T‐cell population. However, they are highly enriched in mucosal tissues including the skin, gut, lung and liver (Table 1). Residing in tissues, Vδ1^+^ T cells adapt to lower nutrient availability and decreased oxygen levels, which is similar to the tumor microenvironment. Incubation in hypoxia *ex vivo* has been shown to enhance γδ T‐cell cytotoxicity. However, tumors in hypoxic environments begin to secrete soluble NKG2D ligands, rendering γδ T cells incapable of killing these cells.^112^ Having previously homed to target organs, adoptively transferred Vδ1^+^ T cells should be capable of homing again to a target organ containing a tumor. Furthermore, protocols have been developed that allow the rapid expansion of highly cytotoxic donor Vδ1^+^ T cells (DOT cells), which are able to control leukaemic cell growth.^57^ These cells acted against a broad range of tumor clones and did not select for resistant strains.^113^ It is thought that this is mediated through innate NK receptors in addition to TCR recognition of tumor cells. Vδ1^+^ γδ T cells express a range of germ‐line‐encoded receptors, which recognise cellular stress (NKG2D) as well as tumor‐ and viral‐associated antigens (NKp44 and NKp46; Figure 1). This is consistent with previous reports, showing that expanded Vδ1^+^ T cells possess broad cytotoxic potential in solid tumors, including colon cancer.^114^ This provides a unique advantage for γδ T cells over conventional αβ T cells. Their ability to recognise a broad range of tumor signals through NK receptors and their TCR allows them to avoid some of the most potent immune evasion mechanisms available to tumors. However, Vδ1^+^ T cells have been poorly characterised in solid tumors. Despite their enrichment in specific organs (Table 1), tumors nonetheless develop in these tissues, indicating many tumors are capable of evading recognition by Vδ1^+^ T cells. γδ T cells may also succumb to inhibition through checkpoint molecules. γδ T cells have been shown to express PD‐1 transiently after activation; however, the expression of PD‐1 and other immune checkpoints such as CTLA‐4, TIM3, LAG3 and TIGIT has been poorly characterised on γδ T cells in human tumors and a combination of these molecules may inhibit TCR and NK‐receptor recognition of tumors.^115^

Vδ2^+^ cells are the majority of circulating γδ T cells in humans, and the vast majority of literature surrounding Vδ2^+^ T cells has focused on a subset expressing the TCR Vγ9 chain. The transcriptional profile of Vγ9^+^Vδ2^+^ T cells appears to be an amalgamation of αβ T cells and NK cells, giving them aspects of both cells’ functions. Vγ9^+^Vδ2^+^ T cells have adaptive features such as a somatic recombination of receptors, memory formation and professional antigen presentation, alongside innate features such as an absence of MHC restriction, recognition of conserved microbial and self‐antigens and ability to perform ADCC.^116^, ^117^, ^118^ A wide range of germ‐line‐encoded activating receptors are also expressed by Vγ9^+^Vδ2^+^ T cells, which are essential for their antitumor function, including NKG2D, which recognise MICA/B.^61^, ^113^ Vγ9^+^Vδ2^+^ T cells have been detected in over 30 solid and haematological malignancies.^109^ In this study, Vγ9^+^Vδ2^+^ T cells were associated with prolonged overall survival in CLL, AML, colon and prostate cancers. Interestingly, Vγ9^+^Vδ2^+^ T‐cell infiltration was independent of αβ T‐cell accumulation, indicating that infiltration by γδ T cells is via a different mechanism to conventional αβ T cells. Vγ9^+^Vδ2^+^ T cells account for about 5% of peripheral blood T cells, so are readily available for *in vivo* and *ex vivo* expansion. The drug zoledronate has been used in several clinical trials to promote the *in vivo* expansion of Vγ9^+^Vδ2^+^ T cells. While this proved a safe treatment, the efficacy was disappointing and failed to prevent progression in most patients.^119^ *Ex vivo* expansion of γδ T cells using zoledronate and IL‐2 has also been trialled in a number of studies, improving disease progression but failing to achieve improved overall survival in a number of solid tumor types (renal cell carcinoma, lung cancer, hepatocellular carcinoma).^119^, ^120^ These early trials should be interpreted with caution as they were designed for assessing safety of γδ T‐cell products and not their efficacy. As of March 2019, there are currently 13 active clinical trials (clinicaltrials.gov) involving the use of γδ T cells to treat a broad range of cancers including leukaemia and breast, pancreatic, ovarian, liver, kidney, lung and brain cancers. These trials involve combinations of *in vivo* expansion using drugs such as zoledronate and alendronate, infusions of *ex vivo*‐expanded γδ T cells and surgical interventions such as cryosurgery or irreversible electroporation (NanoKnife; Table 2). However, these trials utilise techniques used in previous trials with low rates of success. Perhaps then, new approaches to γδ T‐cell‐based immunotherapy are required.

**Table 2:** Ongoing clinical trials involving γδ T cells

Clinical trial ID (NCT)	Disease type	Treatment	Trial phase
*In vivo* expansion			
03862833	Leukaemia	Zoledronic acid+IL‐2	I
01404742	Neuroblastoma	Zoledronic acid +IL‐2	I
00588913	Kidney cancer, lung metastasis	Zoledronic acid + IL‐2 Autologous activated lymphocytes	I/II
02781805	Breast cancer	Alendronate	I
*Ex vivo* expansion			
03533816	AML, CML, ALL, MDS	EAGD T‐cell infusion	I
03183206	Breast cancer	Cryosurgery/IRE + γδ T‐cell infusion	I/II
03183232	Lung cancer	Cryosurgery/IRE + γδ T‐cell infusion	I/II
03183219	Liver cancer	Cryosurgery/IRE + γδ T‐cell infusion	I/II
03180437	Pancreatic cancer	Cryosurgery/IRE + γδ T‐cell infusion	I/II
02418481	Breast cancer	γδ T‐cell infusion	I/II
02425748	Lung cancer	γδ T‐cell infusion	I/II
02425735	Liver cancer	γδ T‐cell infusion	I/II

ALL, acute lymphoblastic leukaemia; AML, acute myeloid leukaemia; CML, chronic myeloid leukaemia; EAGD, expanded/activated γδ T cell; IRE, irreversible electroporation; MDS, myelodysplastic syndrome.

While the current trend in immunotherapy involves the use of checkpoint inhibitors to release the suppression of T cells, this therapy may not drive antitumor responses in innate T cells, such as γδ T cells. As many γδ T cells are not MHC‐restricted, the co‐inhibitory pathways associated with antigen presentation, such as PD‐1 and CTLA‐4, may be redundant in their tumor recognition. Therefore, γδ T cells may require release of additional immune checkpoints such as TIGIT, a potent inhibitor of NK cells.^121^, ^122^, ^123^ The expression of immune checkpoints such as TIGIT, TIM3, LAG3 and NKG2A remains poorly characterised in tumor‐infiltrating γδ T cells and may provide synergistic targets to combine with conventional T‐cell targets such as PD‐1.

Recently, γδ T cells have been incorporated into CAR therapy, producing sufficient cells from Vδ1^+^ and Vδ2^+^ subsets for clinical studies.^124^, ^125^ An additional perquisite of using γδ T cells for immunotherapy lies in their ability to cross‐present processed tumor antigen to αβ T cells, and this process is retained in CAR‐γδ T cells, further enhancing their antitumor effects.^124^

## Future directions

With the advancement of chimeric antigen receptor (CAR) engineering, interest in cellular therapies has increased dramatically. Furthermore, robust expansion protocols for the production of γδ T cells en masse have made their use in the clinic feasible.^32^, ^57^, ^114^ The safety profile of innate lymphocytes compared to conventional T cells and their lack of MHC restriction makes them an attractive target for off‐the‐shelf cell therapy. However, further fundamental research is needed to grasp fully the pleiotropic roles of γδ T cells in cancer. In addition, inhibitory pathways used by tumors to evade recognition by γδ T cells have been poorly characterised and warrant further investigation. Additional and more advanced‐phase clinical trials are required to determine the efficacy of γδ T‐cell‐based therapies. γδ T cells are a strong positive prognostic in most cancers. They naturally infiltrate tissues throughout the body, including lung, liver and the gut, some of the most difficult organs in which to treat malignancies. They recognise a broad range of tumors, not only through their TCR but also through NK receptors. Furthermore, they fail to induce graft‐versus‐host disease and autoimmune complications. This potent effector function, broad range of activity and safety profile make them an ideal potential cellular therapy to enhance current immunotherapy strategies and improve the treatment of solid malignancies.

## Conflict of interest

The authors declare no conflict of interest.
